# Supplementary material for: Empirical methods for controlling false positives and estimating confidence in ChIP-Seq peaks
Source: BMC Bioinformatics. 2008 Dec 5;9:523. doi: 10.1186/1471-2105-9-523 (PMC2628906; doi:10.1186/1471-2105-9-523)
Supplement: Additional File 1 — A variety of html documents from the USeq web site detailing the available applications, their best usage, output file type descriptions, command line menus, etc. [file 1471-2105-9-523-S1.zip › USeqUserGuides/JavaDoc.zip!/JavaDoc/trans/misc/package-summary.html]

trans.misc


|  |  |  |
| --- | --- | --- |
| **Overview** \| **Package** \| Class \| **Use** \| **Tree** \| **Deprecated** \| **Index** \| **Help** | |  |
| **PREV PACKAGE**   **NEXT PACKAGE** | **FRAMES**    **NO FRAMES**     **All Classes** |  |


---

## Package trans.misc

Rough working scripts.

**See:**
  
          **Description**

| **Class Summary** | |
| --- | --- |
| **AverageDoubles** | Slides a 50 index window along an array averaging the contents. |
| **Bar2Gr** | Utility to convert simple one graph xxx.bar files to xxx.gr files. |
| **Bed2Gr** | Converts a bed file to multiple gr files. |
| **BestWindowScoreExtractor** | Finds the best overlapping Window for each region and prints that score with the region information. |
| **CalcGCAndTransform** | Parses out treatment and control oligo info from intervals, calculate GC and transform ratios based on GC corrector, see `TransformRatios`. |
| **CalcGCRatiosFromIntervals** | Parse out treatment and control oligo info from intervals, calculate GC and ratios to look for bias. |
| **CalculateGeneExtensions** | Finds isolated genes that are expressed and using Huber's segmentation algorithm, slices it into three and prints the extension. |
| **ConvertAgilentData** | Reads in an Agilent tiling data file and outputs a tpmap and four cela files. |
| **ConvertBars2TPMapCel** | Reads in multiple directories of bar files and makes xxx.cela and xxx.tpmap files. |
| **ConvertGeoData** | Reads and parses in a GEO data file into a tpmap and xxx.cela files for TiMAT2 processing. |
| **ConvertNimblegenNDF2TPMap** | Converts a Nimblegen NDF txt file to a tpmap.! |
| **ConvertNimblegenPAIR2Cela** | Converts Nimblegen PAIR txt files to a cela files.! |
| **CorrelateGrFiles** |  |
| **Delme** |  |
| **GCRatio** | Container for a ratio and associated %GC content for a particular oligo. |
| **Gr2Bar** |  |
| **GrGraph** | Container for holding info related to a xxx.gr file. |
| **InefficientPseudoMedian** |  |
| **PrependColumn** |  |
| **Regions2Intervals** | Converts binding regions to intervals setting the binding region as the best window and adds 2kb on either side. |
| **ScoredRegionsFormatFixer** | Converts a file format to something usable by Excel from the joinRegionsWithScores.sh script. |
| **Sgr2Bar** |  |
| **SgrComparator** |  |
| **TextPValOrSig2Gr** | Converts a bed file to multiple gr files. |
| **TransformRatios** | Transforms ratio scores in Intervals based on the BAC GC Corrector. |
| **Util** | Static helper methods for all things Affy |
| **Wig2Bar** |  |
| **WindowHistogram** | Makes a histogram of scores from a Window[]. |

## Package trans.misc Description

Rough working scripts.

---


|  |  |  |
| --- | --- | --- |
| **Overview** \| **Package** \| Class \| **Use** \| **Tree** \| **Deprecated** \| **Index** \| **Help** | |  |
| **PREV PACKAGE**   **NEXT PACKAGE** | **FRAMES**    **NO FRAMES**     **All Classes** |  |


---
